# Human cooperation and evolutionary transitions in individuality

**DOI:** 10.1098/rstb.2021.0414

**Published:** 2023-03-13

**Authors:** Cathryn Townsend, Joseph V. Ferraro, Heather Habecker, Mark V. Flinn

**Affiliations:** ^1^ Department of Anthropology, Baylor University, Waco, TX 76798-7334, USA; ^2^ Department of Psychology and Neuroscience, Baylor University, Waco, TX 76798-7334, USA

**Keywords:** human evolution, cumulative culture, cooperation, egalitarianism, private property, transitions in individuality

## Abstract

A major evolutionary transition in individuality involves the formation of a cooperative group and the transformation of that group into an evolutionary entity. Human cooperation shares principles with those of multicellular organisms that have undergone transitions in individuality: division of labour, communication, and fitness interdependence. After the split from the last common ancestor of hominoids, early hominins adapted to an increasingly terrestrial niche for several million years. We posit that new challenges in this niche set in motion a positive feedback loop in selection pressure for cooperation that ratcheted coevolutionary changes in sociality, communication, brains, cognition, kin relations and technology, eventually resulting in egalitarian societies with suppressed competition and rapid cumulative culture. The increasing pace of information innovation and transmission became a key aspect of the evolutionary niche that enabled humans to become formidable cooperators with explosive population growth, the ability to cooperate and compete in groups of millions, and emergent social norms, e.g. private property. Despite considerable fitness interdependence, the rise of private property, in concert with population explosion and socioeconomic inequality, subverts potential transition of human groups into evolutionary entities due to resurgence of latent competition and conflict.

This article is part of the theme issue ‘Human socio-cultural evolution in light of evolutionary transitions’.

Why are we all alone at the pinnacle of the particular direction of rapid evolutionary change that led to… our tendency and ability to cooperate and compete in social groups of millions?

        (Richard D. Alexander [1], p. 1)

## 1. Background

A major evolutionary transition in individuality requires the formation of a cooperative group and transformation of that group into an evolutionary entity in its own right [2]. Human cooperation demonstrates at least some of the features that characterize major evolutionary transitions in individuality ([3–6], see also articles in this issue). It has a complex communication system and fitness interdependence, which in humans takes the form of collective resource acquisition, risk pooling, cooperative breeding and role specialization. Culture—‘socially transmitted information’ [7] or ‘the totality of traditions acquired in a community through social learning’ [8]—was a key selective pressure in human evolution [9–12], and it has been argued that culture is the primary reason for our species' success in the realm of cooperation [13,14]. A hallmark of human culture is widely accepted to be its cumulative capacity, that is, cultural traditions collectively build on one another through modification [8,9,15,16]. Cognitive aptitudes and neurobiological mechanisms for learning, language and sociality underpinned acquisition of information that was increasingly important for hominin survival and reproduction [17–20]. Why humans, and humans alone, evolved such abilities remains an elusive puzzle. Applying theory of transitions in individuality [21,22] may help us to better understand the evolutionary history of human cooperation.

Here we review a series of interdependent transitional stages that we posit were fundamental to the evolution of our extraordinary cooperative capacity. We aim for this model of the evolutionary history of human cooperation to provide insight into the question of whether the human species is undergoing a transition in individuality—where groups of cooperating individuals have become so interdependent that they begin to evolve emergent properties as new higher-level evolutionary entities. Our model has six major components, described below. In the following sections, we evaluate how known hominin traits fit with the model, and infer when and how they would have emerged in several stages. In each stage, we analyse processes that are comparable with two steps in evolutionary transitions in individuality: (1) the formation of cooperative groups, (2) transformation of the group into an evolutionary entity [6].

## 2. Components of the model

(a) Hominin evolution became an increasingly autonomous process wherein hominins gradually became their own most important selective pressure [1]. A key aspect of this process of niche construction is likely to have been cooperative interpersonal relationships among hominin individuals and groups [1,22–24]. We argue that this is plausibly the most important cause of rapid changes in brain evolution, beginning with the origins of the genus *Homo* around approximately 2 Ma [25], that underpin language, cumulative culture and our psychosocial aptitudes.
(b) Crucial to the process of developing cumulative culture and language was a fluid, multilevel, egalitarian sociality similar to that characteristic of modern immediate-return hunter–gatherer societies [26–29]—one that involved flexible, voluntary alliances and cooperative interaction with individuals from other groups (in contrast to chimpanzees and gorillas) and bilateral kinship affiliations [30–34]. We suggest that intra- and intergroup tolerance began to emerge gradually after the split from the last common ancestor (LCA) with panins [35], which contrasts with the hostile inter-community relations of chimpanzees [36,37] that impede cultural transmission.
(c) A flexible pattern of extended family relationships enabled longer periods of child development and increasingly fluid sociality [35,38].
(d) An environment in which cultural innovations and traditions were important for cooperative defence against predators; foraging; egalitarianism; and the evolution of language [39]. Culture depends on communicative strategies for the social transmission of information; the more complex the information, the more sophisticated the requisite strategies need be.
(e) Cumulative culture with ratcheting, or the accumulation of modifications over time [15], created an environment in which social norms became an increasingly powerful influence on human development and behaviour [40].
(f) A key social norm was the creation of rights regarding property ownership. Once private property rights were established, socioeconomic inequalities began to emerge as did major alterations to the physical and social environment. Although the social norms governing private property rights likely first started to emerge during the Late Pleistocene (as evidenced by status artefacts such as grand burials), humans began to drive environmental change more rapidly during the Holocene, modifying energy flow via intensive agriculture, deforestation and emitting greenhouse gases [41,42].

We wish to draw attention to the necessarily incomplete nature of the model that we outline here, due to limitations and uncertainty in the evidence available for reconstruction, and the broad scope of enquiry. Direct evidence of key traits such as cryptic ovulation, protolanguage, ‘mind-reading’, empathy and cooperative defence are unlikely to be found in the archaeological or paleontological record. Inferences, however, may be reasoned from comparative analyses and other sources.

## 3. Discussion

What is so special about the evolutionary trajectory of hominins that resulted in selection for our extraordinary suite of traits? A characteristic feature of mammals is intensive maternal care of altricial offspring. Many mammalian species, including most primates, also have varying levels of alloparenting support and protection by relatives [43]. Beyond these shared features, however, humans also have a suite of highly unusual traits [1]. We are the only species characterized by the *combination* of stable breeding bonds; flexible and extensive alloparenting; male parental effort within multi-male groups; long childhood; cryptic ovulation; extended bilateral and multi-generational kin affiliation; grandparenting; influence of relatives over mate choice; language; variable group composition and inter-group relationships [44,45]. A unique aspect of human sociality that may have been critical for selection for cognitive aptitudes for culture, is the tolerant and strategic nature of intergroup relationships. Mobile hunter–gatherer societies are extensive *multilevel fission–fusion* societies characterized by fluid, open composition and movement of individuals and family units among residential groups [31,–34,46,47]. Information can move among groups of humans far more rapidly than for other hominoids [48,49]. At some point—a first key set of transitions—hominins were able to interact cooperatively with individuals from other groups, and the portal for the viral flow of cultural information began to open. Rapid communication of information would be further enhanced by high mobility enabled by bipedality and a more terrestrial niche. Interactions among fluid groups would have intensified interdependent ratcheting in culture, brains, communication and cooperation [34,50,51], potentiating subsequent transitions in hominin evolution. This hominin pattern stands in contrast to the constricted sociality of chimpanzees, gorillas and orangutans [34,44]; we argue, however, that it has some intriguing similarities to that of bonobos.

### (a) The early transitions- hominin origins

#### (i) 9–4 Ma. Early transitions from last common hominoid ancestor (relative to ***Pan***)

Key traits:

— Increasing terrestriality over the period relative to the last common ancestor (LCA) [52,53]
— Increasing bipedal locomotion [52–55].

Key inferences:

— Increasing mobility and home range.
— Increasing encounters with predators; occasional cooperative defence against predators.
— Increasing ability to carry e.g. infants; food; sticks for protection.
— Increasing intragroup tolerance.
— Simple forms of cooperative breeding (e.g. occasional provisioning of juveniles and lactating mothers).
— Increasing intergroup tolerance (less territorial, flow of cultural information).

A first transition took place at the origins of the hominin taxa in the late Miocene—about 6–9 Ma–when our ancestors split from our shared lineage with chimpanzees and bonobos [52]. The LCA had traits that allowed its several hominoid descendants to evolve in strikingly different ways. We suggest that a key element was a positive feedback loop in selection pressure for enhanced social cooperation initially set in motion by the shift toward terrestrial living (likely related to ecological changes that occurred during the late Miocene) [54]. Although precise dating is difficult to ascertain, such a feedback loop would ultimately come to afford flexible, open social networks with cooperation between groups in hominin evolution. Other elements include a variable, more terrestrial, increasingly open woodland ecological niche, and extended range of mobility.

The increasing terrestriality of early hominins would have increased the risk from terrestrial predators, especially for juveniles and slower-moving females carrying infants. The problem for mothers may have been compounded by gradual changes in hair density [56] and gradual shift to habitual bipedality, which may have been incompatible with the clinging and mounting pattern of infant carrying found among African apes due to hair tensile properties; instead infants may have been increasingly carried in their mother's arms [57,58]. An alternative hypothesis is that as primary dorsal riding in early infancy became more difficult due to the vanished horizontal surface to ride on, infants were positioned initially ventrally and then laterally on the gestational parent (with infant clinging) [59]. Either way, it is likely that some manual support from the mother was required during the first months of infancy, as is the case with gorillas and chimpanzees [59]. The shift to habitual bipedality is estimated to have occurred between 7 and 4 Ma [53].

The risk of terrestrial predators may have been an initial selection pressure for cooperative defence that necessitated foraging in larger groups, but which could diminish foraging efficiency due to within-group feeding competition, as per the ecological constraints model, which also predicts that the further a primate species deviates from the model, the more important are social selection pressures [60]. Diminished efficiency may have imposed a new selection pressure that ratcheted cooperative behaviours, this time for the purposes of innovative cooperative foraging techniques (possibly including culturally transmitted tool use such as sticks or stones for protection, knowledge-sharing of the environment and collective hunting/scavenging of small game). There may also have been situational *need-based transfers* [61] to pool the risk of individual food shortages and stochastic foraging returns. Need-based transfers are a risk-pooling strategy in which resources are transferred to a cooperative partner who is in need without creating debt. There might have been occasional provisioning of juveniles and lactating mothers by other group members.

The cooperative behaviours listed above are plausible based on analogous behaviours in our closest relatives. They suggest that precursor behaviours were either already present in the LCA or that they evolved convergently after hominins split from that lineage, but early hominins would have had additional pressure to form defensive cooperative groups due to the risk from terrestrial predators. The defence that chimpanzees use against leopards [62,63] suggests the feasibility of hominins cooperatively defending group members, and male kin may have been important in this. The use of sharp sticks by chimpanzees [64] suggests the potential for similar behaviour by early hominins. Cooperation may have been crucial to support the nutritional needs of juveniles and mothers with infants. Collective hunting occurs in chimpanzees [65] and bonobos [66]. Food sharing occurs in chimpanzees and bonobos, but sharing among unrelated individuals appears to occur more often in bonobos, with female bonobos in particular sharing with other females and with infants, though male bonobos also share with females [67]. Female bonobos have been observed engaging in food sharing across communities [68]. Similar behaviour occurs in female vampire bats, which engage in food transfers to needy non-kin associates, a behaviour that may be co-opted from maternal care [69].

It has been suggested that social monogamy emerged as early as in *Ardipithecus ramidus* [70]. Such behavioural traits are difficult to infer from the available evidence—indeed, body size dimorphism in early hominins undermines the argument for monogamy [71,72]. There are, however, significant neurobiological differences between humans and other hominoids in the striatum (a subcortical area of the brain that contributes to prosocial behaviours) [70]; as well as in other brain areas [73,74] that suggest early evolution of social cooperation. The early forms of cooperative breeding may have centred on female kin rather than social monogamy. Cooperation between mother and juvenile and a grandmother and her daughter can be favoured by natural selection regardless of the breeding system due to high coefficients of relatedness and low opportunity costs [75,76].

It is difficult to ascertain from the fossil record what capacity early hominins had for culture, and whether there was diffusion across communities. While it has been suggested that strontium from teeth shows that *Paranthropus robustus* and *Australopithecus africanus* had philopatric social systems similar to those of chimpanzees [77], it is not currently possible to determine conclusively residence or migration patterns, nor the extent of cooperative intergroup relationships, from fossilized teeth [78–80]. The behaviours of extant primates, however, offer some clues. Chimpanzees, gorillas and orangutans do not have intergroup tolerance among males. Bonobos and lowland gorillas are less hostile, but lowland gorillas do not evidence cooperative interaction that would facilitate widespread social transmission of information—culture. Chimpanzee communities are isolated by their lethal hostility to outsiders [36], with minimal opportunities for information to move between communities [81]. Nonetheless, some cultural diffusion does appear to occur through the emigration of postpubertal females from their natal groups into neighbouring groups [82]. While bonobos have less aggression between groups than do chimpanzees, they appear to have comparatively limited use of tools, which at first glance might suggest that bonobos have less culture [83].

Both chimpanzees and bonobos have fission–fusion communities divided into multi-male, multi-female subgroups with male philopatry and female emigration; hence it seems likely that the LCA had similar social organization [21,84]. Chimpanzee females spend much of their time alone because there is female competition over foraging resources [85] and because they are at risk of being targeted for infanticide [86,87]. There is emerging evidence of cooperation between unrelated bonobo females: forming strong affiliations, foraging together in large mixed groups, sharing food and engaging in coalitional food defence against males [88]. High female gregariousness is also found among *Pan troglodytes verus* [87]. Female bonobos have been observed to cooperate across communities, forming coalitions with outgroup members to attack a common target [88]. Female bonobos may also form strong bonds with male bonobos, particularly mother–son relationships [83]. By contrast, in chimpanzees, males cooperate to hunt and patrol borders [36].

Although tool use appears less prevalent among bonobos than chimpanzees, cultural diffusion of tool use across bonobo communities has been documented, with more similar tool-use repertoires among geographically proximate populations than more distant ones [89]. Furthermore, tool use is not the only indicator of culture; the vocalizations, social grooming gestures, sociosexual behaviours and prey preferences of bonobos show intriguing indications of cultural transmission, and crucially, cultural diffusion across overlapping communities [84,90–93]. Slight differences in neurobiology between bonobos and chimpanzees may enable more prosocial behaviours and hence facilitate cooperative culture, in bonobos [94].

The unique evolutionary trajectory of hominins may have favoured enduring social bonds that occasionally crossed over community lines. There are cultural behaviours in bonobos that, if present in the LCA, could have been precursors to the emergence of fluid sociality. Bonobos show highly empathic behaviour across communities, e.g. food sharing [68] and cases of females adopting infants from outgroups with no aggression from the adoptive communities [95]. Cluster analysis of *Pan* social systems indicates that there is a clear ingroup/outgroup distinction in bonobo social organization, with tolerance toward outgroups [96]. In combination with high mobility facilitated by bipedality in increasingly open woodland settings, pro-sociality could ultimately lead to more flexible tolerance between communities, and the potential for the fluid multilevel networks that characterize mobile hunter–gatherer societies to begin emerging (although modern forms most likely first appeared in the transition to *Homo*) [49].

In terms of theory of evolutionary transitions in individuality, this stage of hominin evolution corresponds to the first step: the formation of a cooperative group, which requires an ecological/efficiency benefit to cooperation and a mechanism for directing the benefit to the cooperator [6]. We have outlined three possible ecological benefits of cooperation at this stage: i. predator defence (a benefit to all group members but especially mothers and juveniles); ii. increased foraging efficiency and risk pooling, and iii. rudimentary forms of cooperative breeding (a benefit to mothers and juveniles). While i. and ii. would directly benefit all cooperators in a group, all three benefits would be especially important for mothers and their juvenile offspring.

We have discussed two mechanisms that would help to ensure that the benefits of cooperation to mothers would outweigh the risks. First, cooperation between mothers and their offspring and between grandmothers and daughters could be favoured through kin selection in the absence of social monogamy (although most of these cooperative relationships are between females, bear in mind the cooperation between mothers and their sons in bonobos). Second, we suggested that the risk pooling function of need-based transfers may be particularly important for mothers and juveniles, using the sharing behaviours of female bonobos and female vampire bats as an analogy. Agent-based modelling of need-based transfers shows them to be particularly beneficial in situations where networks are modular and small [97]. Female cooperative breeding coalitions and their offspring could form a needs-based network because they have a common interest in risk pooling, where nursing mothers are supported with resource transfers of food and time/energy spent looking out for predators.

The next transitional stages present a striking contrast to the relatively slow evolutionary pace of the first stage. For instance, there is little change in brain size over this initial lengthy period, nor evidence of major anatomical changes other than the shift to bipedalism.

#### (ii) 4–2 Ma. gracile Australopithecine***s /*** early ***Homo*** grade

Key traits:

— Expansion of cerebral cortex [98].
— Inhabiting a greater range of environments including increasingly open settings [99].
— Continued adaptation to terrestrial niche [52].
— Habitual bipedal locomotion [53].
— Reduced canine size and dimorphism, but continued body size dimorphism [72].
— Dietary changes as evidenced by reduced anterior dentition; reduced thick enamel on teeth, orthognathic face and protruding nose [100].
— Stone tools [101].
— Heavier, secondary altricial infants [102–104].

Key inferences:

— Social foraging; increasing food sharing.
— Increasing alloparenting.
— Resolution of infanticide.
— Incorporation of males into alloparenting networks.

Expansion of the cerebral cortex was already in process 4 Ma [98]. It has been inferred from crania that *Australopithecus afarensis* infants had protracted brain growth relative to the other hominoids [103]. *Australopithecus* also had infants significantly heavier than chimpanzee offspring; carrying them may have limited arboreality, causing increased vulnerability to predators and further selection for alloparenting behaviours among females and possibly males [102]. Pelvic reconstructions and fetal head sizes imply that australopithecines gave birth to immature, secondary altricial newborns [104]. If this is the case, australopithecine mothers must have carried and attended to heavier, more dependent infants for longer infancies than is typical for primates.

Birthing of secondary altricial infants would present considerable challenges to mothers in a social world in which infanticide from conspecifics is a continuous threat; there may have been selection pressure on females to develop counter-strategies to resolve infanticide during this phase. It has been suggested that social monogamy with paternal care in primates is a defence against male infanticide because both parents can play a defensive role and biparental care enables a shortened lactation time [105]. It is possible that hominins would have begun a shift to more stable long-term breeding bonds with paternal care during the transition in the terminal point of this phase. An alternative possibility is that the infanticide problem was resolved via a similar route to that taken by bonobos.

Male-led infanticide in bonobos is absent or rare despite having traits found in those species in which males benefit from infanticide [106,107], which may be the result of strong female coalitions, or paternity confusion e.g. cryptic ovulation, and non-lethal aggression in males [83,84,107]. Female coalitions, which are also present in humans, may have already been present in the LCA or they may have emerged during the early phases of hominin evolution. Cryptic ovulation in humans takes a different form from in bonobos, but may have evolved as a defence against infanticide in addition to facilitating stable breeding bonds and reducing intragroup mate competition and conflict [108,109].

Neurologically immature infants would have required considerable assistance, suggesting cooperative care and provisioning [104]. Female coalitions may have played an alloparental role in aspects of infant care such as cooperative foraging, defence against predators, food sharing, infant monitoring and cultural transmission of information between communities and generations, as temporal and energetic demands on mothers carrying and caring for large-bodied altricial infants would have been considerable. Furthermore, environmental instability in the Plio-Pleistocene is suggested to be linked with key adaptations in hominins including behavioural innovations [110]. The Shifting Heterogeneity Model [99] suggests that the tropical African environment that shaped the early stages of human evolution was one in which ecosystems were constantly fragmented and reassembled, causing subtle and intricate selective pressures and incremental evolutionary change. A likely behavioural innovation may have been alloparenting, as unstable environments are predicted to correlate with alloparental care based on comparisons with other alloparental species and non-industrialized human societies in differing ecologies [111,112].

While persistent sexual size dimorphism in *Australopithecines* [72] suggests continued high levels of mate competition among males, it does not necessarily follow that paternal care of infants was absent. Comparative evidence from owl monkeys, baboons, Assamese macaques, mountain gorillas and chimpanzees suggests that paternal investment does not depend entirely on the existence of exclusive pair bonds and high paternity certainty, but could emerge from features of behaviour present in chimpanzees, such as long-term male–female associations and food sharing [113].

If males were incorporated into alloparental cooperative networks in early grade *Homo* species, a new feedback loop in selection could have been set in motion, where male parental effort (e.g. defence and food provisioning) could have a high pay-off to individual male fitness by increasing the survival of vulnerable infants, followed by sexual and social selection of cooperative males by females and ultimately by mixed networks of alloparental cooperators—an emergent form of the flexible multilevel societies that characterize modern human hunter–gatherer societies. Human male parental behaviour is strikingly different from that of chimpanzees, and evidenced in neurological differences [74].

Reorganization and evolution of the sensor motor and temporal lobes of the brain in *Australopithecine* to members of early *Homo* were likely crucial in expanding memory and cognitive functions [114]. Prevalence of Oldowan stone tools from around 2.6 Ma marks the start of a phase transition in which natural selection was likely replaced as the main mechanism of evolution by a process of niche construction where already moderately advanced capacity for cognition and vocal communication was accelerated [98]. A brain region related to human language, Broca's area, was already in place in *Homo habilis.* [115]. *Homo* *naledi* shared the inferior frontal gyrus (contains Broca's area) and lateral orbital gyrus with humans, suggesting that these new structures in the prefrontal cortex were ancestral within the genus *Homo* [116]. The lateral orbital gyrus is important for social cognition [117].

In terms of theory of transitions in individuality, this stage corresponds with the first step: the formation of a cooperative group [6]. We infer widening of female cooperative breeding networks that emerged in the previous stage. Benefits to cooperators would have been defence against predators and infanticidal males, more efficient foraging, and need-based transfers to risk-pool against stochastic food shortages. Primate infants are particularly susceptible to food shortages due to lower fat reserves and weaker muscle tissue [118], which suggests that infants needed transfers from their mothers and their mothers' cooperative partners (e.g. female coalition partners, male mating associates and older offspring). Females and infants could also benefit from female alloparenting coalitions and paternal care to prevent infanticide. Males could obtain a reproductive benefit from protecting infants if they recognized their own offspring due to mating partnerships with specific females or by protecting full or even half siblings—provided the cost was not too high. Thus males could have gradually been incorporated into female cooperative alloparental networks, leading to growth of networks and extension of need-based transfers to cope with food shortages outside of alloparenting contexts.

Need-based transfers consisting of relaxed territoriality to allow sharing of bountiful resource patches with individuals from needy neighbouring groups could have caused greater intergroup cooperation, thus providing a social environment conducive to cultural diffusion of learned behaviours and technology. Bonobo females appear to have cooperative relationships across groups, as discussed above, therefore intergroup cooperation likely emerged from female kin relationships. Stone tool technology towards the end of this stage supports the inference that extension of cooperative networks between groups was in motion.

### (b) The middle transitions—becoming human

#### (i) 2 Ma –700 ka. *Homo ergaster*/*erectus*

Key traits:

— Increasing brain size with high metabolic cost [119,120]. Incipient frontal lobe reorganization around 1.76 Ma; human-like frontal lobe organization from approximately 1.5 Ma [121].
— Acheulean manufactured tools from 1.76 Ma [122].
— Out of Africa and the peopling of Eurasia [123].
— Increasing body size, especially in females [124]; decreasing sexual dimorphism [125].

Inferences:

— Cognitive and neural advances including those that support tool use and protolanguage [98].
— Potential capacity for core cumulative cultural evolution [126] (see [18] for ‘core criteria’).
— Social learning, complex imitation, emulation, teaching [19,126,127].
— Increasingly altricial young.
— Stable long-term breeding bonds or social monogamy.
— Extended bilateral family networks.
— More complex communication, including gradual cultural development of a primitive semiotic ‘protolanguage’ [128].

The early Pleistocene was a period of dramatic changes [129]. Hominins developed Acheulean stone tools [130] and began to spread into Eurasia [123]. Hominins were becoming increasingly ‘ecologically dominant’ (i.e. the importance of social selection was increasing relative to Darwin's traditional hostile forces of nature), in concert with the emerging importance of the social environment as a selective pressure on brain evolution [1,131–133]. While some of the behavioural manifestations for cumulative culture that are evidenced in the archaeological record occur in the middle Pleistocene in Africa [18,133–135], the roots of Alexander's ‘suite’ of traits—including brain enlargement, protolanguage, empathy, cryptic ovulation, flexible nested coalitions etc.—may have started earlier, perhaps beginning to co-evolve in the transition to *Homo ergaster/ erectus* [18,136,137].

The most rapid acceleration rate of encephalization in hominin evolution occurs from approximately 2.1 Ma [68]. Another rate change (slower) occurs from approximately 1.49 Ma [120]. Endocranial shape evidence indicates that there were changes in brain organization, with incipient frontal lobe reorganization from approximately 1.76 Ma and the emergence of modern human-like frontal lobe organization from approximately 1.5 Ma [121]. Encephalization had a high metabolic cost [119].

The social brain hypothesis [131], in which bigger brains were required to manage complex social relations in cooperative groups of primates, is supported by correlations between group size and brain indices [138], but has some limitations [139,140]. It has been suggested that ‘cognitive benefits that do not directly improve an organism's energy balance can only be selectively favoured when they produce such large improvements in reproduction or survival that they outweigh the negative energy effects of a large brain’ [140]. Rapid encephalization during this stage may be related to extending kinship-based cooperative networks, and innovating new strategies to transmit culture such as semiotic communication with simple syntax. Adaptations to complex cooperation could have contributed to better tool use, foraging efficiency and risk-pooling strategies, thus increasing quantity and stability of caloric intake for individuals.

A major adaptive transition, the extended family—including life-long sibling relationships, multigenerational relationships and affinal relationships [44,45]—may have further enhanced tolerance and connections among hominin groups. Decreasing sexual dimorphism [125] suggests that long-term stable breeding bonds or social monogamy became prevalent. Egocentric bilateral kinship recognition could have created flexible intergroup networks that opened new channels for cultural diffusion [31,34,141]. Extensive intergroup kin networks would accelerate the role of cooperative interactions with others as a selective pressure [1,132,142]. We suggest these changes in kinship underpinned subsequent transitions in cooperation, cumulative culture, language and the technology that becomes evident later in the archaeological record in the Middle Stone Age [134].

The origins of human language are difficult to determine with precision [143], but it may have begun to emerge very slowly through processes of cumulative cultural acquisition once the capacity for premodern language had been reached—associated with early *Homo erectus sensu lato* about 2 Ma [128]. The transmission of information related to the manufacture of Acheulean stone tools may have benefitted from protolanguage with simple syntax [98]. It has been argued that ‘collective processes of cultural exploration produced new behaviours, which in turn changed individual cognition in ways that were eventually partially genetically accommodated’, an innovative cultural process of language construction that would have involved high levels of trust and cooperation [144]. Once the brain had the capacity for premodern language, it would require a positive feedback loop between cultural innovation and genetic accommodations to build up to the capacity for modern language. It is possible that during this phase a primitive semiotic code was elaborated to better coordinate ever-more complex cooperation.

The reorganization of the frontal lobe from approximately 1.76 Ma to approximately 1.5 Ma may indicate culturally led evolution related to innovations in communication that made the brain ‘language ready’ [121]. The rate of encephalization slowed by about 1.49 Ma but a steady rate, independent of body size, continues throughout the Pleistocene [120]. Continued brain size increase is consistent with the ecological dominance–social competition model [1,132,142,145].

In terms of transitions in individuality, this stage corresponds to the ongoing process of forming a cooperative group; but we also start to see evidence of the second step: the transformation of the group into an integrated entity via the emergence of communication that coordinates cooperation at the group level, as well as some suppression of reproductive competition [6]. Stable long-term breeding bonds or elementary social monogamy could form the basis of bilateral kinship networks that connected cooperative breeding networks together to form larger social networks spanning across residential groups, incorporating both sexes and extending the sphere of cooperation beyond alloparenting. Protolanguage may have emerged in part to transmit information about individuals' reputation for cooperation because cheating is a problem for cooperative systems (i.e. an individual benefitting from cooperation but not contributing to its cost). Reproductive competition may have been somewhat suppressed at this stage by a system based largely on social monogamy and limited polygyny.

#### (ii) 700 ka - 40 ka Archaic *Homo*

Key traits:

— Changes in life history [146].
— Further decrease in sexual dimorphism [72].
— More complex technology; including prepared cores and hafting (134).
— Full capacity for cumulative cultural evolution (see [16] for the extended criteria).
— Widespread habitual control of fire from approximately 400 ka [147].

Key inferences:

— Compositional language involving social scenario building, detailed management of social reputations, humour etc.
— Emergence of social norms and social identity management in concert; social institutions such as marriage.
— Increasingly sophisticated material culture imbued with value; emergence of property rights.
— Increasingly complex systems of sharing and helping [148].
— Non-competitive egalitarian social organization including sharing the recognized property of others [26–29].
— Unusual sexual characteristics, including cryptic ovulation [108,109].
— Increasing foresight, planning, theory of mind, mental time travel; associated with areas of the prefrontal cortex [149,150].
— Social emotions such as guilt, embarrassment, pride, restraint and humour.
— Increasing creativity and innovation [151,152].
— Variable patterns of kinship. Extensive cooperative breeding, involving parents, grandparents, siblings, other kin and friendships [43].
— Social monogamy but variable, gender egalitarianism in postmarital residence [33].
— Ability to forge social ties (friendships, trading relationships and marriages) beyond local demes.

The cultural process of building language itself was likely the first form of cumulative culture. The capacity for compositional language (the ability to combine discrete symbolic units) may have emerged in the merging species *late Homo erectus s.l.* and *pre-archaic Homo sapiens* [128]. From this point, it became cognitively possible for language involving social-scenario building and detailed management of social reputations to begin to emerge through cultural construction. Language may have evolved in part for ‘negotiating group activities or transmitting and enforcing social norms’ [153]. Clearly language is an extraordinary social adaptation that expands the potential for the flow of information and cumulative culture; there is, however, no well-accepted archaeological evidence of cumulative culture prior to the Middle Stone Age ca 300 ka. This is not in itself evidence against the emergence of semiotic communication well before the clearest archaeological evidence of cumulative material culture, because language evolution may have involved a ratcheting effect that began with managing social relationships. Indeed, the core cognitive skill that is thought to lead to the emergence of cumulative culture is transmission fidelity due to accurate imitation, which can be supported by language and teaching [126].

The decrease in sexual dimorphism during the middle Pleistocene [72] is consistent with changes in sociality, family relationships and gender egalitarianism [29,33]. Archaic *Homo* and eventually, anatomically modern humans [72], were on a distinctive evolutionary trajectory involving increasingly sophisticated tool kits and exploitation of diverse environments including northern and high-altitude regions with frigid climates.

Kish Bar-On & Lamm [154] suggest that social norms and social identity negotiation are intertwined features of human behaviour that likely emerged in concert as early as *H. heidelbergensis* [155]. It is likely that social norms such as conflict avoidance were put to use in creating non-competitive egalitarian social structure and suppressing intra-group male–male competition, hence the decrease in sexual dimorphism [29]. Modern non-competitive egalitarian societies have a set of social norms and related practices that function to maintain social equality, known as levelling mechanisms e.g. moving to avoid conflict [26]. There is also a set of levelling mechanisms that disengage people from the potential that property ownership has to create socioeconomic differentiation between people. Exclusive land tenure rights or the ownership of environmental resources are strongly discouraged in such societies [26]. Egalitarian practices are ‘antithetical to the production of prestige items or wealth for personal uses' [156]. The relatively complex material culture and lithic technology of the latter half of this phase and relatively quick pace of technological change [133] suggest that cumulative culture was in full operation. It also suggests the emergence of social norms governing the use of property.

By property, we mean more than an individual's sense of possession over a resource or respect for possession of resources, which is clearly present in other species [157]. Property here refers to resources that are recognized by others as belonging to a specific individual or group because they are the recognized yield of their labour [27], where recognition involves social reinforcement of norms related to possession. In modern egalitarian hunter–gatherer societies, the social norms related to property function to systematically disengage people from the potential that it has to cause inequalities e.g. sanctions on the accumulation of property, pressure to share and redistribution of possessions [26].

The latter end of this stage includes a transitional phase that saw the emergence of proto *Homo sapiens* in Africa and subsequent development of modern human traits such as complex language that likely included kinship terminologies [158] and universal kinship [141], leading to more expansive cooperative networks, fast-paced cumulative culture, specialized technologies and perhaps nascent symbolic representations in material culture (e.g. engraved shells from Java that date to approx. 0.54 Ma [159]). Widespread controlled use of fire across the Old World from 400 ka suggests sufficient tolerance between hominin sub-populations for cultural diffusion to cross wide regions in a short space of time [147]. Gendered division of labour likely emerged in order to facilitate childcare, but this can be flexible [160].

Direct fossil or archaeological evidence for unusual traits of human sexuality such as cryptic ovulation and menopause is lacking; our inferences are based on the fit with a cluster of changes in other traits, such as diminished sexual dimorphism and life history that included an extended childhood [1,45]. Similar reasoning applies to inferences of complex cognitive and emotional aptitudes such as self-awareness, embarrassment and mental time-travel, which would have been crucial for the establishment of egalitarian norms and that likely became increasingly important in the complex sociality of *Homo sapiens* [20,150,161].

In terms of transitions in individuality, this stage corresponds to the expansion of the cooperative group and concomitant expansion of communication to coordinate cooperation [6]. Keeping track of the cooperative reputations of others would be required [131] for cooperative individuals to avoid exploitation by free riders. A potential problem with language is that it can itself be used to deceive or cheat, as signals are cheap rather than costly, thus it is dependent on cooperative intersubjectivity [162]. This suggests that initially there must have been substantial common interest between senders and receivers, for example, high levels of relatedness or aligned reproductive interests [6], which is concordant with cooperative networks consisting of alloparents and extended family.

This stage also involves suppression of conflict, especially male–male reproductive competition through egalitarian social norms such as respect for monogamous pair bonds, conflict avoidance and levelling mechanisms against property accumulation. The transition to facultative or obligate eusociality has only taken place in other organisms when strict lifetime monogamy ensures that offspring who aid parents benefit their full siblings [6]. Monogamy in humans is not strict, and there is persistence of moderate sexual dimorphism. Nonetheless, long-term pair bonds may have been sufficient to favour concealed ovulation and male parenting supported by hormonal responses [108,109,163].

### (c) The later transitions—explosion of cultural diversity

#### (i) 300 ka – 12 ka. Modern *Homo sapiens*

Key traits:

— World-wide geographical distribution and population growth [164,165].
— Brain size already within the range of present-day humans by 300 ka; brain shape reached present-day variation by 100 ka—35 ka [166].
— Extinction of remaining ancestral species or side branches; apparent limited adaptive radiation in *Homo* [167] despite rapid evolutionary changes.
— Explosive increases in cumulative culture, diversity of information [168,169].
— Increasingly specialized technologies for foraging/hunting, resource storage [169].
— Cave paintings; representational figurines from approximately 40 ka.

Inferences:

— Flexible, strategic and bilateral kinship.
— Cosmological belief systems with ritual performance.
— Ethnically distinguished societies with potential for inter-cooperation.
— Increasing population density with population pressure in some areas.
— In critical contexts, emergent private property rights are established over fixed resources, e.g. salmon runs; key hunting territories; fresh water sources in desert areas; some storage.
— Sporadic and seasonal sedentarization.
— Increasingly variable social organization and inequality [170], delayed-return hunter–gatherer societies [26].

Anatomically modern humans are associated with rapid increases in cultural and niche diversity [171]. The human brain evolved to its greatest complexity [17,134]. Human life history shifted to longer childhoods and longer lives with menopause, potentiating social learning and family relationships including grandparenting [172]. Flexible universal kinship emerged [141], including extended bilateral, affinal and multi-generation links and ultimately adaptive flexible social responses to different environments [173].

Complex technologies, symbolic representations and behaviours seemingly dependent on cumulative culture became ubiquitous over the broad, nearly worldwide geographical spread of humans. Changes in brain and neuroendocrine mechanisms that underpin our most extraordinary human cognitive aptitudes—self-awareness, consciousness, empathy, imagination, social scenario building—and our complex emotions—attachment, guilt, embarrassment—appear to have been increasingly selected for during this period of intense sociality [18,134,174]. There is evidence that the oxytocin gene, a neuroendocrine system responsible for bonding and attachment in mammals, underwent positive, and balancing, selection in humans since our split from *Pan* [175,176].

The term ‘anthropocene’ refers to an epoch of human impacts on the global environment, often associated with rising concentrations of trapped methane and carbon dioxide in the atmosphere due to human fossil fuel use [177]. Early anthropogenic impacts include extinction of mammal species beginning as early as 50 ka in Australia; deforestation and soil erosion related to the rise and spread of agriculture, and estimates of rising methane and carbon dioxide emissions beginning several thousand years ago [178]. Rapid population growth, beginning in the Late Pleistocene, is a common factor that helps to explain anthropogenic transformations such as climate change and declining biodiversity. The human cultural niche was well-established by this time, and emergent social norms, such as private property, likely facilitated population growth. The earlier phases of the *Homo* genus appear to have been characterized by at least one population bottleneck, with an estimated effective population size of only 18 500–26 000 human ancestors living at 1.2 Ma [164]. An approximately 10-fold increase in the rate of population growth began at about 36 ka in the Late Pleistocene [165], which is after the expansion of *Homo sapiens* into Europe as early as 48 ka with a population as small as approximately 1500 people connected to one another in what is suggested to be a functional social network of low population densities maintained by social norms [179]. Between 44 and 35 ka, Neanderthals in Europe became extinct possibly due to competition with or absorption by *Homo sapiens* [180,181].

The Late Pleistocene population increase occurs during a period associated with complex and diverse material culture, new technologies and likely role specialization [171], e.g. a surgical amputation of a limb dates from approximately 31 ka in Borneo [182]. We also see the emergence of relatively sedentary hunter–gatherer societies with emergent inequality in resource-rich areas. For example, there is evidence of permanent architectural structures that would have taken considerable time and energy to construct, such as the circular mammoth bone features of Upper Palaeolithic sites with storage pits in Eastern Europe, from 25 ka to 12 ka [183–185]. Storage, exchange networks, prestige items, grand burials and permanent architecture are associated with private property (meaning that the owner/s have exclusive rights), and with the potential for socioeconomic inequalities. Political alternations between hierarchical and egalitarian possibilities have been interpreted as ‘an emergent property of human societies in the highly seasonal environments of the last Ice Age’ [170].

In terms of transitions in individuality, this stage is noteworthy for the ongoing evolution of communication and the emergence of role specialization beyond gender, which may have heightened fitness interdependence. In modern egalitarian hunter–gatherer societies people have free and equal access to wild resources, and they can therefore go about obtaining their own requirements without dependencies on specific others [26]. They rely on each other for learning skills, sharing resources and knowledge, childcare, and protection from wild animals, but to an extent it is possible for people to detach themselves without economic deprivation. In most of the modern world, individuals are more economically dependent on others; e.g. a place to live usually requires purchase or rental of real estate. In the late Pleistocene, in highly seasonal environments, we also infer the apparent rise of social norms granting private property rights, which is likely to have initiated a resurgence of resource competition.

#### (ii) 12 ka–present. The Domesticated World

— Worldwide distribution of *Homo sapiens.*
— Population explosion [165,186,187].
— Bottleneck in Y-chromosome [188–190].
— Domestication of plants and animals [191].
— Social hierarchies, unilineal societies, ranked societies.
— Resource and reproductive competition between patrilineal descent groups [192].
— Increased capacity for storage (food, tools, fuel, materials).
— Warfare increases in contexts of sedentism, hierarchy, and population pressure.
— New forms of socioeconomic organization such as big man societies, chiefdoms, and early state formation with feudal systems and monarchies; colonial empires; democracies; communism; capitalism and the global market economy.
— Explosion of information technologies, from writing to computers.
— Anthropogenic transformation of the adaptive landscape, e.g. declining biodiversity, mass migrations, pandemics, climate change, global warfare, and nuclear weapons.

The past 10 ka have seen a 1000-fold increase in population size [165]. From 8 to 4 ka in Africa, Asia, Europe and the Middle East there was a strong bottleneck in the Y-chromosome in which the effective population size of women is up to 17-fold that of men, which is consistent with cultural transmission of fitness, i.e. changes in social organization that increased variance in the offspring number of men [188–190]. It has been suggested that the cultural change involved was a widespread switch to patrilineal kin groups and competition between these groups [190], a switch that may have come about due to agriculture, private property rights, and emergent political complexity [191–194]. At approximately 4–3 ka, a rate increase has been calculated to have occurred with much faster exponential, or super-exponential population growth [186,187]. A key cultural shift that may have caused demographic changes was the proliferation of social norms that recognize private property rights. The accumulation of private property usually accompanies sedentarization, which requires storage, and it has the potential to heighten reproductive competition in men due to differential property ownership and polygynous marriages that favour the reproductive success of wealthier men, as found in sedentarized hunter–gatherer societies, big man societies, and chieftanships [195].

Prior to the adoption of agriculture, wealth inequalities were likely limited to a few sedentary societies of hunter–gatherers, such as those of the Pacific Northwest Coast [196]. Sedentarization and the creation of private property rights over resources may be the result of either agriculture (which involves storage, agricultural tools, other people's labour, and land tenure) or foraging practices that rely on fixed resources e.g. salmon runs. Storage usually emerges as a response to seasonality and unpredictably [197]. Once established, it potentiates the emergence of inequality because it effectively creates property that can be used by an individual or clique to gain political leverage over others, and it supports greater population density and sedentary aggregations [197–199].

The emergence of agriculture, which began about 12 ka, appears to have played a role in increasing population growth (through fertility increases associated with sedentarization and cultivation) [200], possibly in a boom-and-bust pattern [201]. In order for agriculture to emerge as a novel subsistence strategy, it required a critical mass of hunter–gatherers to adopt changes in social norms that led to privatization of resources and failure of the levelling mechanisms that disengage people from property accumulation [202–204]. Once farming was adopted by a critical mass, groups of farmers would ultimately out-reproduce foragers due to fertility increases, thus spreading the farming and private property cultural package [203].

Primary state societies gradually began emerging from about 5.5 ka, likely as the result of territorial expansion of chieftanships, which required bureaucratization of central authority and additional resources to maintain [205]. Cooperative societies have subsequently expanded their reach, whether that be chiefdoms absorbing neighbouring groups, or states cooperating to create new organizations of governance or trade (e.g. the United Nations), or large multinational corporations that wield considerable political power. Expansions may involve warfare and takeovers or voluntary association. Profound political and economic inequalities within and between societies have emerged.

In terms of evolutionary transitions in individuality, the key dynamic at play during the Holocene is a dramatic surge in intragroup and intergroup competition, related to unequal access to resources. This amounts to a change in social norms, which instead of repressing competition became repurposed to allow property accumulation and economic inequalities to gain hold and to proliferate. We see individuals, especially men, using wealth and status to increase their inclusive fitness at the expense of others, resulting in resource and reproductive competition between patrilineal descent groups [192] to an extent that is discernible in the Y-chromosome bottleneck between 8–4 ka [188–190]. Intragroup competition is usually a barrier to the transition of a group to an evolutionary entity [6] but there is also ambiguity about the boundaries of potential entities due to the multilevel nature of human societies, their fission–fusion qualities and the multiple social identities of human individuals.

## 4. Conclusion

Let us return to Richard Alexander's question, *why are humans the only species who cooperate and compete in social groups of millions?* [1]. Human groups are multilevel societies composed of nested and flexible coalitions that may include families and kin groups but often unrelated individuals with distinct reproductive interests [206]. Hominin evolutionary history, since the split from the LCA with panins, is characterized by the increasing use of culture to solve problems cooperatively. Our early hominin ancestors encountered intense selection pressures in the natural environment that were resolved with cooperation. When selective challenges were met with innovative and novel communication strategies that afforded the social transmission of ever-more complex information, hominins entered into an evolutionary niche involving ratcheting of culturally influenced cooperative strategies. Humans, and humans alone, evolved through culturally coordinated cooperation that has profoundly altered the global environment and changed the adaptive landscape for ourselves and other species.

Human cooperation demonstrates at least some of the features that characterize major evolutionary transitions in individuality. It has a complex and novel communication system (cumulative culture and language) and widespread fitness interdependence (in the form of collective resource acquisition, risk pooling, cooperative breeding and role specialization). However, intragroup competition and conflict are far from negligible in large-scale cooperative human societies, despite the fact that they have mechanisms for suppressing ‘cheating’ (e.g. culturally mediated social norms and institutions) [207], and despite the fact that large cooperative groups can be formidable competitors.

The social and political organization of cooperative human groups can vary enormously—from the most egalitarian, where resource and reproductive competition is more effectively suppressed, to societies with growing economic inequality and private property rights. The social norms that govern human cooperation are also highly variable. Norms are socially constructed by people with a large body of shared cultural information, and they are therefore responsive to differing social environments. While the most egalitarian hunter–gatherer societies have social norms that compel extensive sharing and sanctions on the accumulation of property, modern nation-states or multinational corporations uphold private property rights and allow profound wealth inequalities between individuals. Hunter–gatherer egalitarianism is a system of cooperation in which competition for resources is suppressed to a much greater degree, and consequently there is less reproductive conflict. Before private property, human societies might have been approaching the state of what has been termed ‘egalitarian organisms', which are evolutionary entities in which individual members are not close genetic kin but achieve a high degree of cooperation by means of mutualism [208].

Private property rights may have emerged through latent competition as opportunistic individuals subverted egalitarian levelling norms, therefore the emergence of societies with private property rights suggests a turn away from what might have been a process in which human groups became evolutionary entities of the ‘egalitarian organism’ kind. Nonetheless, societies with private property rights have high levels of interdependence and are able to maintain a cooperative basis through centralized governance.

In the modern world, individuals may have complex social identities that effectively make them a part of more than one social group, sometimes even competing groups. The fission and fusion of large social networks may occur in response to perceived competition from other groups (e.g. the creation of the African Union in 2002) or perceived competition from within the group (e.g. the withdrawal of Great Britain from the European Union in 2020). We suggest that competition between groups and multilevel fission–fusion processes are permutations that are worthy of further analysis in terms of evolutionary transitions in individuality.

## Data Availability

This article has no additional data.
